# Prevalence and Determinants of Chronic Pain Post-COVID; Cross-Sectional Study

**DOI:** 10.3390/jcm11195569

**Published:** 2022-09-22

**Authors:** Panagiotis Zis, Christiana Ioannou, Artemios Artemiadis, Katerina Christodoulou, Stefania Kalampokini, Georgios M. Hadjigeorgiou

**Affiliations:** 1 Medical School, University of Cyprus, Nicosia 1678, Cyprus; 2 Second Department of Neurology, School of Medicine, Attikon University Hospital, National and Kapodistrian University of Athens, 12462 Athens, Greece; 3 Department of Neurology, Nicosia New General Hospital, Nicosia 2029, Cyprus

**Keywords:** COVID-19, chronic pain, neuropathic pain, long COVID

## Abstract

Introduction: Chronic pain is increasingly recognized as part of long COVID syndrome, mainly in the form of myalgias. However, chronic pain has several forms, and according to our clinical experience, COVID-19 survivors suffer from numerous painful syndromes, other than myalgias. The aim of our study was to estimate the prevalence of chronic pain, describe the commonest painful syndromes and identify pain determinants in a random population of COVID-19 survivors. Methods: This was a cross-sectional study conducted at the Medical School, University of Cyprus. A random population of 90 COVID-19 survivors was recruited. Demographic and COVID-19 related clinical characteristics were recorded. The painDETECT and DN4 questionnaires were used to evaluate the painful syndromes. Results: The prevalence of chronic pain was estimated to be 63.3%. The most common site of pain was low back (37.8%), followed by joints (28.9%) and neck (12.2%). Patients with chronic pain compared to subjects without pain were older (50.5 ± 15.9 versus 42.2 ± 12.6, *p* = 0.011) and more likely to be female (71.9% versus 45.5%, *p* = 0.013). One in six subjects (16.7%) reported new-onset pain post COVID-19. The prevalence of neuropathic pain was estimated to be 24.4%. After adjusting for age and gender, headache during COVID-19 was a statistically significant predictor of neuropathic pain, increasing 4.9 times (95% 1.4–16.6, *p* = 0.011) the odds of neuropathic pain. Conclusion: Chronic pain—especially neuropathic—is widely prevalent in COVID-19 survivors. One in six subjects will develop new-onset pain that will persist beyond the acute phase of the disease and, therefore, should be considered a symptom of long COVID syndrome.

## 1. Introduction

Neurological symptoms associated with SARS-CoV-2 disease (COVID-19) have been reported early on in the pandemic with anosmia being one of the first to be reported, occurring in the acute phase of the disease [1]. The rapidly increasing number of COVID-19 cases allowed numerous observations of neurological diseases associated with COVID-19 during the acute phase, including cerebrovascular events, encephalopathies, myelopathies and Guillain–Barre syndrome [2].

Though we are hopefully moving closer to the end of this pandemic, it has been observed that many COVID-19 survivors have been suffering from symptoms that persist long after their infection with SARS-CoV-2. Long or post COVID syndrome, is the collective term to denote such persistence of symptoms [3]. According to the World Health Organization, the most common symptoms of post COVID-19 syndrome include fatigue, respiratory difficulties, memory, concentration or sleep problems, loss of smell or taste, depression and anxiety. Chronic pain has also been part of the long-COVID syndrome, mainly in the form of myalgias. However, chronic pain has several forms and can be broadly distinguished to nociceptive and neuropathic. Our clinical experience shows that COVID-19 survivors often suffer from the latter, which is defined as pain caused by a lesion or disease of the somatosensory nervous system [4].

The aim of our study was to estimate the prevalence of chronic pain in general and neuropathic pain in particular, describe the commonest painful syndromes and identify pain determinants in a random population of COVID-19 survivors.

## 2. Material and Methods

### 2.1. Procedure and Participants

This was a cross-sectional study conducted at the Medical School, University of Cyprus. COVID-19 survivors were recruited following a call for volunteers through social media. To be enrolled, the patients had to meet the following inclusion criteria: (1) a definite diagnosis of COVID-19, (2) age equal to or greater than 18 years and (3) able to provide written informed consent.

The study protocol was approved by the Cyprus National Bioethics Committee (reference 2020/64).

### 2.2. Measures

Demographic characteristics included age, gender and body mass index (BMI).

COVID-19 related data were recorded: date of diagnosis, means of COVID-19 diagnosis and neurological symptoms during the disease (namely, anosmia/hyposmia, ageusia/hypogeusia, fatigue, headache, dizziness and other, if applicable).

Presence of pain persisting for more than 4 weeks was questioned with all participants during recruitment. The painDETECT questionnaire [5] was used to map the location(s) of pain. For each location, pain was further evaluated via the DN4 questionnaire [6]. DN4 is a clinician administered screening tool consisting of 10 yes/no items. A score of equal to or greater than 4 was considered as diagnostic of neuropathic pain [7]. When pain was present, the participant was asked whether this occurred post COVID-19 or whether it was present from before.

During the interview, all participants were asked about their past medical history.

### 2.3. Statistical Analyses

A database was developed using the Statistical Package for Social Science (version 28.0 for Mac; SPSS). Frequencies and descriptive statistics were examined for each variable. Comparisons between groups were made using Student’s *t*-tests for normally distributed continuous data, Mann–Whitney’s *U* test for non-normally distributed and chi-square test for categorical data. For multivariate analyses, logistic regression models were used. All accuracy and generalization assumptions for the model were checked. Level of significance was set at the 0.05 level.

## 3. Results

### 3.1. Study Population

Ninety COVID-19 survivors (62.2% female) with a mean age of 47.5 ± 15.3 years (ranging from 18 to 89 years old) met the inclusion criteria and were recruited to this study. The participants suffered COVID-19 between February 2020 and May 2021. The mean time between COVID-19 and recruitment to the study was 189 ± 105 days.

The most frequent neurological symptom during COVID-19 was fatigue, affecting 49 subjects (54.4%) followed by anosmia/hyposmia (47.8%), ageusia/hypogeusia (47.8%), headache (44.4%) and dizziness (12.2). Seventy-six (84.4%) subjects suffered from at least one neurological symptom during COVID-19.

Table 1 summarizes the demographic and clinical characteristics of our study population.

### 3.2. Prevalence and Determinants of Chronic Pain

**Figure 1:**
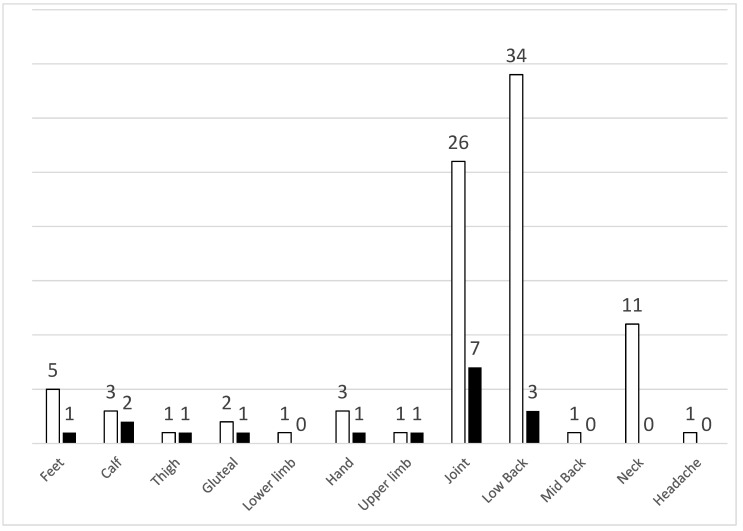
Distribution in sites of pain and absolute numbers in all subjects with pain (white columns) and new-onset pain only (black columns).

Fifty-seven (63.3%) subjects suffered from chronic pain at recruitment. As illustrated in Figure 1, the most common site of pain was low back (37.8% of the total population), followed by joints (28.9% of the total population) and neck (12.2% of the total population).

Univariate analysis showed that patients with chronic pain, compared to subjects without, were older (50.5 ± 15.9 versus 42.2 ± 12.6, *p* = 0.011) and more likely to be female (71.9% versus 45.5%, *p* = 0.013). Both age and gender remained statistically significant determinants of pain after the logistic regression analysis.

Table 2 summarizes the demographic and clinical characteristics of patients with and without chronic pain and Table 3 presents the results of the multivariate analysis.

### 3.3. Prevalence and Determinants of Neuropathic Pain

Table 4 summarizes the demographic and clinical characteristics of patients with neuropathic and non-neuropathic pain. Twenty-two patients (24.4%) suffered from neuropathic pain. The commonest site of neuropathic pain was low back, accounting for 9 patients (10%), followed by neck (*n* = 4), joint (*n* = 4), feet (*n* = 2), thigh (*n* = 1), lower limbs (*n* = 1) and hands (*n* = 1).

Univariate analysis showed that patients with neuropathic pain were significantly more likely to have suffered from headache during COVID-19 compared to patients with non-neuropathic pain (63.6% versus 31.4%, *p* = 0.017).

As shown on Table 5, multivariate analysis showed that after having adjusted for age and gender, headache during COVID-19 remained a statistically significant predictor of neuropathic pain, increasing the odds of neuropathic pain by 4.9 times (95% CI 1.4–16.6, *p* = 0.011).

### 3.4. Prevalence and Determinants of New-Onset Pain Post COVID-19

Fifteen participants (16.7%) reported new-onset pain post COVID-19. As illustrated in Figure 1, the most common site of the new-onset pain was joints (7.8%) followed by low back (3.3%). Univariate analysis showed that patients with new-onset pain post COVID-19 were significantly more likely to be female compared to patients with no pain (80% versus 45.5%, *p* = 0.025). Table 6 summarizes the demographic and clinical characteristics of patients who developed pain post COVID-19 compared to COVID-19 survivors without pre- or post-COVID pain.

## 4. Conclusions

Population estimates for the prevalence of chronic pain vary widely according to case definition and ascertainment methods, and time, place and population. However, in a recent review of the literature, Mills et al. reported that chronic pain might affect up to 50% of the population [8]. Our cross-sectional study demonstrated that more than three out of five COVID-19 survivors experience chronic pain, a percentage higher than expected. Female gender and increasing age correlated with the presence of chronic pain in this population, which is in keeping with the literature [8].

Another interesting finding was that one in six COVID-19 survivors experienced new-onset pain that became chronic. This can possibly explain the higher-than-expected prevalence of chronic pain in our study population. Female gender was significantly associated with experiencing new-onset chronic pain post-COVID.

Regarding neuropathic pain, Yawn et al. carried out an epidemiological study that estimated the community prevalence of neuropathic pain to be almost 10% [9]. In our study, we showed that almost one out of four COVID-19 survivors experience neuropathic pain, which is also higher than expected. An interesting observation in our population was that after having adjusted for gender and age, the strongest predictor of neuropathic pain was the presence of headache during COVID-19. As all neurological symptoms that the patients experienced during the acute phase of the disease were recorded during their participation in this study, we opted not to use diagnostic tools to assess the type, characters and intensity of the headache, due to the potential recall bias. Whether headache during the acute phase of COVID-19 can be a predictor for the symptoms experienced by patients, subsequent development of chronic neuropathic pain remains to be confirmed in larger prospective studies.

Our results should be interpreted with some caution given the limitations of our design. Firstly, although our study population has been random with a wide range of ages, it consists of a moderate number of subjects. Secondly, our study group was infected between February 2020 and May 2021 and, therefore, have not been exposed to the omicron variant. Further studies are needed to confirm whether our findings are applicable to subjects who suffered COVID-19 due to the omicron variant.

In conclusion, chronic pain—especially neuropathic—is widely prevalent in COVID-19 survivors. One in six subjects will develop new-onset pain that will persist beyond the acute phase of the disease and, therefore, should be considered a symptom of long COVID syndrome.

## Figures and Tables

**Table 1:** Demographic and clinical characteristics of the total study population (*n* = 90). SD, standard deviation; BMI, body mass index.

	Total Population (*n* = 90)
**Demographics**	
Age, in years (SD)	47.5 (15.3)
Female gender (%)	56 (62.2)
BMI	26.8 (4.6)
**COVID-19 neurological symptoms**	
Anosmia/Hyposmia (%)	43 (47.8)
Ageusia/Hypogeusia (%)	43 (47.8)
Fatigue (%)	49 (54.4)
Headache (%)	40 (44.4)
Dizziness (%)	11 (12.2)
Any neurological symptom (%)	76 (84.4)

**Table 2:** Demographic and clinical characteristics of COVID-19 survivors with and without chronic pain. SD, standard deviation; BMI, body mass index.

	Chronic Pain (*n* = 57)	No Chronic Pain (*n* = 33)	*p*
**Demographics**			
Age, in years (SD)	50.5 (15.9)	42.2 (12.6)	0.011
Female gender (%)	41 (71.9)	15 (45.5)	0.013
BMI	27.3 (4.9)	26.0 (3.7)	0.205
**COVID-19 neurological symptoms**			
Anosmia/Hyposmia (%)	29 (50.9)	14 (42.4)	0.439
Ageusia/Hypogeusia (%)	28 (49.1)	15 (45.5)	0.737
Fatigue (%)	30 (52.6)	19 (57.6)	0.650
Headache (%)	25 (43.9)	15 (45.5)	0.883
Dizziness (%)	7 (12.3)	4 (12.1)	0.982
Any neurological symptom (%)	48 (84.2)	28 (84.8)	0.936

**Table 3:** COVID-19 survivors’ characteristics investigated for their association with chronic pain. CI, confidence intervals. * Adjusted odd ratios (OR) presented.

Variable	OR * (95% CI)	Wald	*p*-Value
Age (per year)	1.044 (1.01–1.079)	6.358	0.012
Female gender	3.359 (1.308–8.625)	6.339	0.012

**Table 4:** Demographic and clinical characteristics of COVID-19 survivors with neuropathic and non-neuropathic pain. SD, standard deviation; BMI, body mass index.

	Neuropathic Pain (*n* = 22)	Non-Neuropathic Pain (*n* = 35)	*p*
**Demographics**			
Age, in years (SD)	54.1 (12.4)	48.3 (17.6)	0.098
Female gender (%)	17 (77.3)	24 (68.6)	0.477
BMI	26.9 (5.0)	27.5 (4.9)	0.550
**COVID-19 neurological symptoms**			
Anosmia/Hyposmia (%)	12 (54.5)	17 (48.6)	0.661
Ageusia/Hypogeusia (%)	13 (59.1)	15 (42.9)	0.233
Fatigue (%)	14 (63.6)	16 (45.7)	0.187
Headache (%)	14 (63.6)	11 (31.4)	0.017
Dizziness (%)	3 (13.6)	4 (11.4)	0.805
Any neurological symptom (%)	21 (95.5)	27 (77.1)	0.065

**Table 5:** COVID-19 survivors’ characteristics investigated for their association with neuropathic pain. CI, confidence intervals. * Adjusted odd ratios (OR) presented.

Variable	OR * (95% CI)	Wald	*p*-Value
Age (per year)	1.036 (0.997–1.076)	3.217	0.073
Female gender	1.784 (0.471–6.747)	0.727	0.394
Headache	4.910 (1.449–16.636)	6.532	**0.011**

**Table 6:** Demographic and clinical characteristics of COVID-19 survivors with new-onset pain compared to COVID-19 survivors without pain. SD, standard deviation; BMI, body mass index.

	Post COVID Pain (*n* = 15)	No Pain (*n* = 33)	*p*
**Demographics**			
Age, in years (SD)	48.7 (14.3)	42.2 (12.6)	0.100
Female gender (%)	12 (80.0)	15 (45.5)	0.025
BMI	29.2 (6.1)	26.0 (3.7)	0.136
**COVID-19 neurological symptoms**			
Anosmia/Hyposmia (%)	8 (53.3)	14 (42.4)	0.482
Ageusia/Hypogeusia (%)	8 (53.3)	15 (45.5)	0.613
Fatigue (%)	11 (73.3)	19 (57.6)	0.296
Headache (%)	9 (60.0)	15 (45.5)	0.350
Dizziness (%)	1 (6.7)	4 (12.1)	0.566
Any neurological symptom (%)	13 (86.7)	28 (84.8)	0.869
